# Diet, physical activity and socio-economic disparities of obesity in Lebanese adults: findings from a national study

**DOI:** 10.1186/s12889-015-1605-9

**Published:** 2015-03-21

**Authors:** Marie Claire Chamieh, Helen J Moore, Carolyn Summerbell, Hani Tamim, Abla Mehio Sibai, Nahla Hwalla

**Affiliations:** Department of Nutrition, Faculty of Agriculture and Food Sciences, American University of Beirut, PO Box 11-0236, Riad El-Solh, Beirut, 1107-2020 Lebanon; School of Medicine, Pharmacy and Health, Durham University, Stockton-on-Tees, UK; Department of Internal Medicine, American University of Beirut Medical Center, Beirut, Lebanon; Department of Epidemiology and Population Health, Faculty of Health Sciences, American University of Beirut, PO Box 11-0236, Riad El-Solh, Beirut, 1107-2020 Lebanon

**Keywords:** Obesity, Prevalence, Diet, Physical activity, Socioeconomic status, Adults, Gender, Lebanon

## Abstract

**Background:**

The prevalence of obesity within countries varies by gender, age, lifestyle and socioeconomic factors. Identification of behavioural factors that are associated with obesity within the country’s context is critical for the development of effective public health programs which aim to prevent and manage obesity. The objective of this study was to assess age and gender differentials in the prevalence of obesity in Lebanon and examine correlates of obesity with a focus on socioeconomic disparities.

**Methods:**

Following the WHO STEPwise guidelines, a national survey was conducted in Lebanon in 2008–2009. Households were selected randomly from all Governorates based on stratified cluster sampling method. One adult aged 20 years and over was randomly selected from each household for the interview. Anthropometric measurements and 24 hour recall dietary intake were obtained. The final sample included 1244 men and 1453 women. Descriptive statistics were computed for BMI, waist circumference, and percent body fat. Multivariate logistic regression analysis was carried out to assess the relationship between energy intake and obesity adjusted for relevant co-variables.

**Results:**

The prevalence of obesity among Lebanese adults was 26.1%. Gender differences in obesity estimates were observed across age groups and the three obesity classes, with men showing higher prevalence rates at the younger age groups (20–49 years), and women showing higher prevalence rates in older age groups (50 years and above). Obesity showed significant associations with socio-economic status in women; it decreased with higher educational attainment (OR = 0.54, 95% CI: 0.32, 0.91), greater household assets (OR = 0.26; 95% CI: 0.10, 0.72) and lower crowding index (OR = 0.62; 95% CI: 0.39, 0.98), net of the effect of other co-variates. There was a significant positive association between obesity and energy intake in both genders, and a negative association between obesity and physical activity, significantly among women.

**Conclusion:**

Lifestyle and socioeconomic determinants of obesity are identified in this Lebanese population. Policy makers and service providers need to tailor public health strategies to tackle obesity accordingly.

## Background

The pattern of the obesity epidemic over the past few decades has varied across countries and among population subgroups within countries [1]. This variability is greater in developing countries experiencing nutrition transition, and is partly the result of a higher pace of urbanization, modernization and affluence as well as a concomitant dramatic shift in dietary habits and lifestyles [2]. Particularly rapid rises in prevalence rates of obesity have been noted in Brazil, Mexico and Sub-Saharan Africa [3], and relatively high estimates rivalling those of the United States are documented in affluent Arab counties such as Kuwait (42%) and Qatar (40%) [4]. Lebanon, a small middle–income country in the Arab region, has been classified by the World Health Organization as being in the early stages of nutrition transition, falling within the same category as other intermediate per capita income countries such as Egypt, Jordan, Syria, Libya, and Morocco [5]. With a population estimate of around 3.7 million [6], Lebanon is characterized by a high urbanization rate (87%) and high migration flows from rural to urban settlements [7].

Research on obesity and its determinants in Lebanon was confined to small-scale surveys conducted on selected segments of population groups and restricted to specific regions. A comprehensive national study that dates back to 1997 [8] showed high obesity prevalence rates in the adult population aged 20 years and older, in both men and women (14% and 19% respectively). Low education, non-smoking, and family history were found to be positively associated with obesity [8]. A smaller subsequent study also noted elevated levels of metabolic syndrome and abdominal obesity in a sample of Lebanese adults drawn from various health centres in the country [9].

In response to the growing need for country-level trends in chronic disease risk factors, including overweight and obesity, a second national survey was conducted in 2008–2009 by the same investigators of the 1997 study, using similar procedures and data collection methods [10] . Compared with the 1997 study, authors reported a significant two-fold increase in the prevalence of obesity in both the paediatric and the adult populations [10]. Researchers also reported a high prevalence of an energy-dense western dietary pattern replacing the traditional plant-based Lebanese diet, in the same study population [11].

Using the data set of the 2008–2009 study, we explore the distribution of the obesity classes (I, II, and III) among the Lebanese adult population by gender and age groups, and we also examine correlates of obesity with a focus on gender, lifestyle and socioeconomic factors.

## Methods

### Study design

The data for the present study were obtained from the national cross-sectional Nutrition and Non-communicable Diseases Risk Factor (NNCD-RF) survey conducted in Lebanon between May 2008 and April 2009. The study sample was based on the sampling frame provided by the National Survey of Household Living Conditions, which was conducted by the Ministry of Social Affairs/Central Administration of Statistics in collaboration with United Nations Development Programme (UNDP), and which covered primary residences across the Lebanese territory [6].

The sample was drawn from randomly selected houses based on stratified cluster sampling. The strata were the Governorates of Lebanon comprising the totally urban capital Beirut and five other governorates (Mount Lebanon, North, Beqaa, Nabatiye, and the South) that are a mixture of rural villages and urban cities. Clusters were further selected at the level of districts (urban and rural areas), and housing units constituted the primary sampling units. Using the Kish-method [12], one adult aged 20 years and older was randomly chosen from each household (excluding pregnant and lactating women and individuals with mental disabilities). Recruitment efforts targeted a sample with an age, sex, and governorate as well as district distribution proportional to that of the Lebanese population [6], and the final sample was self-weighted.

Given that BMI is the main outcome for the study, sample size was calculated based on the previously estimated adult obesity prevalence of 17% [8] considering a power of 0.8, a confidence interval of 95%, and an error margin of ± 1.5.

The study followed the World Health Organization (WHO) STEPwise Survey guidelines for data collection [12]. All data, reported and measured, were collected at the participant’s house by face-to-face interviews using a comprehensive questionnaire adapted from the WHO Survey. This provided information on socio-demographic characteristics (age, sex, marital status, education, work status, and income) and behavioural factors including smoking, diet, meal pattern (main regular meals consumption, snacking, and eating out), and physical activity pattern. The short version of the International Physical Activity Questionnaire (IPAQ) was adopted as an interviewer-administered questionnaire to assess physical activity [13,14]. Family history of obesity (including parents, brothers and sisters, grandparents, aunts, and uncles) was also recorded. Anthropometric measurements were taken using standardized techniques and calibrated equipment. The average value of two measurements was adopted for the study.

Intensive training on study instruments and data collection procedures, as well as field methodology pre-testing were executed prior to the actual field work. Field monitoring was performed throughout the study to maintain the quality of the data collected. The study was conducted according to the guidelines laid down in the Declaration of Helsinki; the original study design and protocol were approved by the Institutional Review Board of the American University of Beirut, and informed consents were obtained from all subjects.

### Measures

#### Anthropometric measurements and definitions of overweight and obesity

Height, weight, skinfold thickness, and waist circumference were measured based on standardized techniques. Subjects, in light indoor clothing and with bare feet or stockings, were weighed to the nearest 0.1 kg by use of a calibrated balance (Seca 877Germany). Height was measured with participants barefoot by use of a stadiometer (Seca 213). It was recorded to the nearest 0.5 cm. Body mass index (BMI) was calculated as the ratio of weight (Kg) to the square of height (m). Obesity was defined according to the World Health Organization standardized criteria [15]. Individuals with a BMI ≥30 Kg/m^2^ were considered obese, and obesity was further categorized into class I obesity (BMI = 30.0 to 34.9 Kg/m^2^), class II obesity (BMI = 35.0 to 39.9 Kg/m^2^), and class III obesity (BMI ≥40 Kg/m^2^).

Waist circumference (WC) was measured at the midpoint between the lower costal border and the iliac crest [16] by use of a plastic measuring tape to the nearest 0.5 cm (Seca 201). Using a skinfold calliper (LANGE Beta Technology Inc., Maryland), skinfold thickness measurements were performed on the right side of the body and at four sites (biceps, triceps, subscapular, and suprailiac) to the nearest 0.1 mm. Percent body fat (% BF) was then computed from the sum of the four measured skinfolds according to the Durnin and Womersley formula [17]. The sex-specific cut-off points of 25% and 32% body fat were used to indicate adiposity in men and women, respectively [18]. Waist circumference measurements ≥ 94 cm and ≥80 cm for men and women, respectively, were used to indicate abdominal obesity [19].

#### Dietary assessment

Food consumption data was obtained during the face-to-face interview using the 24-hour recall instrument administered by trained nutritionists and following the 5-step multiple-pass method [20]. Particular consideration was given to factors that maintain the quality of the reported dietary data, such as respondent reactivity, non-directive probing, and portion size estimation [18,21]. To help subjects in assessing the portion/amount of food consumed, quantification tools recommended by the 24-hour recall protocols (www.csrees.usda.gov) were used. These included standard measuring cups and spoons, household measures, as well as food photos and food models exemplifying the most commonly consumed foods in frequent serving sizes.

To report estimates of energy and macronutrients that fulfil the objectives of this study, the Nutritionist Pro software (N-squared Computing Nutritionist IV. Silverton, OR: N-squared Computing; 1995) was used for the analysis of the participants’ 24-hour recalls. Its database was expanded and adapted to population-based food intake surveys previously carried out in Lebanon [8,22]. A major amount of these nutrient values was obtained by chemical analyses of foods and popular mixed dishes in Lebanon and the Middle East, carried out at the American University of Beirut–Lebanon [23]. Other values were added by comparison with a similar food in the database. During this study, locally consumed foods from standardized traditional Lebanese recipes collected by the interviewers throughout the field work were also added to the software database for nutrient analysis, thus preventing the loss of detailed description of a certain cultural food by pre-coded recipes (priori coding). Estimates of energy and macro-nutrient intakes were then computed and exported into the SPSS software for analysis.

#### Indicators of socioeconomic status

Three proxy indicators for socio-economic status were used in this study. These included ownership of household assets [24], the crowding index, and educational attainment [25]. Although information on income (Lebanese Pounds) was collected during the face-to-face interviews, it was not included in the analysis because of the large number of missing cases. For the current study, availability of household assets was designated by a composite score of 7 which was created based on the count of essential household assets listed in the interview questionnaire: fridge, washing machine, oven, television, DVD player, air-conditioner, computer, in addition to ownership of a mobile phone and a vehicle. The analysis also used the American Crowding Index, a proxy measure of socioeconomic status applied in both industrialized and non-industrialized countries. It is calculated as the number of people living in a household per number of rooms available in the house (excluding kitchen and bathrooms). An index > 1 indicates an overcrowded household with few economic resources [25]. Education is often used as a proxy measure of socioeconomic status in epidemiological studies [25]. In the current study, education was measured as a categorical variable where participants were asked to specify their highest level of educational attainment (primary, high school, higher education diplomas and degrees).

#### Statistical methods

The survey yielded an acceptable non-response rate of 10% and a sample size of 2697 participants, with an age and gender distribution proportional to that of the baseline population [26]. Subjects with missing anthropometric measurements (n = 39) and subjects identified as underweight (n = 50) were excluded, yielding 2608 cases available for the analysis. Descriptive statistics were computed for BMI, waist circumference (WC), and percent body fat (%BF). Prevalence of obesity, elevated waist circumference, and elevated percent body fat was determined, and data were presented for both genders categorized into 10-year age groups, with the last age category including all those ≥70 years. Difference in mean BMI, WC, and %BF across gender was tested using *t*-test, and difference in prevalence of obesity, elevated waist circumference, and elevated percent body fat across gender was tested using Chi-Square. Mean intakes of total energy (SD) and percent of energy from macronutrients were computed after exclusion of subjects who reported that their 24-hour recall was not representative of their habitual intake (n = 68), yielding a sample size of 2540 subjects. To further improve the general quality of the dietary data in this study, evaluation of the validity of reported energy intake was carried out using the revised Goldberg Method [27,28]. A total of 602 subjects were identified as implausible reporters of energy intake (424 under-reporters and 178 over-reporters, data not shown). These were later excluded from the specific analysis of the associations between the independent variables and the dependent outcome (BMI). Multivariate logistic regression analysis was then carried out with obesity (BMI ≥ 30 kg/m^2^) as the dependent variable and a number of potential co-variates as the independent variables. All co-variates were entered into the logistic models simultaneously, allowing the predictive ability of each variable to be assessed whilst controlling for all others. The criteria for inclusion of co-variates in the final model were statistical significance at the bivariate level and theoretical importance of the variable as associated with obesity based on the literature. The final multivariate model included socio-demographic characteristics as well as indicators of socioeconomic status: age, marital status, household assets, crowding index, and education. Total energy consumption and proportion of energy intake from macronutrients (fat, protein, and carbohydrates) were divided into tertiles and included in the model as categorical variables. Intake of daily regular meals (consistent meal consumption) was also integrated in the model. Three levels of physical activity expressed as MET-minutes/week were computed according to the IPAQ scoring guidelines [14] and assigned to the study population: low (negligible physical activity), moderate (minimal level of physical activity), and high (health-enhancing physical activity). Depending on frequency distribution, physical activity was entered into the model as a two-category variable: low level versus moderate to high level.

Prevalence odd ratios (ORs) and their 95% confidence intervals (CI) were calculated to estimate the association between obesity and the various co-varieties in both genders. Given the variability in the association between the co-variates and obesity among younger and older adults, analysis was also stratified by age groups (20–59 years and ≥60 years). A P-value of <0.05 was considered significant and all analysis was conducted using SPSS version 19.

## Results

### Study sample

Of the study participants, 46.8% were males and 53% were females, representing the national ratio of male to female population [6]. This study sample of 2608 adults aged 20 years and over was comparable to the target population of residents in Lebanon, in terms of demography and geography [6,8,26]. As shown in Table 1, celibacy (never married) was higher in men than women (38.6% and 25.3%, respectively), and educational attainment was comparable between both genders at all levels. Smoking was higher among men than women (68.5% and 50.1%), and a higher proportion of men reported low physical activity (48% vs. 43%), whereas more women were moderately active (34% vs. 28%). More women than men reported skipping one or more meal per day (56.2% vs. 49.5%, P < 0.001). Total mean energy consumption was 7024KJ (SD 3660) for the adult female population ≥20 years and 10273KJ (SD5603) for the adult male population ≥20 years. Mean consumption of energy from fat was at the higher limit for both men 36.8% (SD 10.1) and women 37.5% (SD 11.1), whereas mean percent energy consumption from carbohydrates was at the lower recommended cut-off: 48.2 (SD 11.4) and 49.2 (SD 11.8) for men and women, respectively [29].

**Table 1:** **Socio-demographic, lifestyle, anthropometric, and dietary characteristics of study sample ≥20 years (n = 2608)**

**Variable**	**Men (n = 1222)**		**Women (n = 1386)**		
	**Age (years) ≥20**				
	**Socio-demographic characteristics**				
	**%**	**n**	**%**	**n**	P-Value*
**Governorates**					
Capital (Beirut)	12.6	159	9.2	128	.002
Other	87.4	1062	90.8	1258	
**Marital status**					
Single	38.6	471	25.3	351	.000
Married	58.1	709	62.5	865	
Divorced, separated, widowed	3.4	41	12.2	169	
**Educational**					
Preliminary or less	22.0	269	20.6	286	.612
Complimentary	25.3	309	24.3	336	
High school/Diploma	24.1	295	26.0	360	
University	28.6	349	29.1	404	
**Household assets**					
<7 items	44.8	544	57.5	792	.000
≥ = 7 items	55.2	669	42.5	585	
**Crowding index**					
CI < 1 person/room	39.5	481	35.7	492	.042
CI ≥ 1 person/room	60.5	736	64.3	888	
**Family history of obesity**					
No	61.9	757	54.2	746	.000
Yes	38.1	463	45.8	636	
	**Lifestyle characteristics**				
	**%**	**n**	**%**	**n**	P-Value*
**Smoking**					
No	31.5	385	49.9	692	.000
Yes	68.5	837	50.1	694	
**Physical activity**					
Low	48.0	570	43.4	591	.009
Moderate	28.5	338	34.0	463	
High	23.5	279	22.6	307	
**Meals pattern**					
Skips 1 or more meals a day	49.5	604	56.2	778	.000
Eats regular 3 meals a day	50.5	615	43.8	606	
	**Anthropometric characteristics**				
	**Mean**	**SD**	**Mean**	**SD**	P-Value*
**Weight (kg)**	82.5	15.5	68.5	14.2	.000
**Height (cm)**	173.1	7.4	159.2	6.4	.000
**BMI (Kg/m** ^**2**^ **)**	27.5	4.7	27.1	5.8	.048
**Waist circumference (cm)**	94.9	12.9	86.5	14.3	.000
**% Body fat**	26.4	6.8	35.6	6.3	.000
	**Dietary characteristics**				
	**(n = 1193)**		(**n = 1347)**		
	**Mean**	**SD**	**Mean**	**SD**	P-Value*
**Energy (Kjoules)**	10273	5603	7024	3660	.000
% Fat	36.8	10.1	37.5	11.1	.000
% Protein	14.8	5.0	14.5	5.2	.000
% Carbohydrate	48.2	11.4	49.2	11.8	.000

*Significant at p < 0.05.

*Differences across gender were examined using *t*-test and chi-square.

The mean BMI for the total sample population was 27.5 (SD 4.7) for men and 27.1 (SD 5.8) for women (Table 1). Mean estimates of waist circumference (cm) were 94.9 (SD 12.9) for men and 86.5 (SD 14.3) for women, and those of percent body fat were 26.4 (SD 6.8) and 35.6 (SD 6.3) for men and women respectively. All anthropometric mean estimates exceed the healthy cut-offs for both genders.

The overall prevalence rate of obesity in the sample was 26.1% (Table 2). Obesity prevalence did not differ significantly between men and women (26.4% and 25.9%, respectively, P = 0.89). Nevertheless, the distribution of obesity across the age groups showed a clear gender differential with men showing higher prevalence rates at the younger age groups (20–49 years), and women showing higher prevalence rates in older age groups (50 years and above). These gender and age differentials in obesity prevalence rates were most apparent in obesity class II (35.0-39.9 Kg/m^2^) and obesity class III (40 kg/m^2^) to the extent that women were more likely than men to show class II and class III obesity (P < 0.05).

**Table 2:** **Prevalence (%) of obesity among a sample of the adult Lebanese population (n = 2608) in 2009 by age and gender**

	**Age groups (years)**						
	**20-29**	**30-39**	**40-49**	**50-59**	**60-69**	**70** ^**+**^	**Total**
	**n = 750**	**n = 630**	**n = 495**	**n = 310**	**n = 230**	**n = 193**	**n = 2608**
	**Obese (BMI ≥ 30 Kg/m** ^**2**^ **)**						
Men	16.8	23.5	31.7	38.2	32.4	33.0	26.4
Women	8.1	21.0	28.7	48.7	45.7	52.4	25.9
Both genders	12.1	22.1	30.0	43.5	39.7	41.3	26.1
	**Class I obesity (BMI 30.0 to 34.9 Kg/m** ^**2**^ **)**						
Men	13.4	17.4	24.8	28.9	28.8	30.0	21.1
Women	6.9	16.0	15.2	26.6	27.8	27.7	16.2
Both genders	10.0	16.7	19.4	27.7	28.3	29.0	18.5
	**Class II obesity (BMI 35.0 to 39.9 Kg/m** ^**2**^ **)**						
Men	2.5	3.9	4.1	9.2	2.9	3.6	4.1
Women	1.3	3.7	8.3	16.5	12.7	15.7	6.9
Both genders	1.9	3.8	6.5	12.9	8.3	8.8	5.6
	**Class III obesity (BMI ≥ 40 Kg/m** ^**2**^ **)**						
Men	1.1	2.1	2.8	0.0	1.0	0.0	1.4
Women	0.5	1.7	5.1	5.7	6.3	9.6	3.4
Both genders	0.8	1.9	4.0	2.9	3.9	4.1	2.5

**Figure 1:**
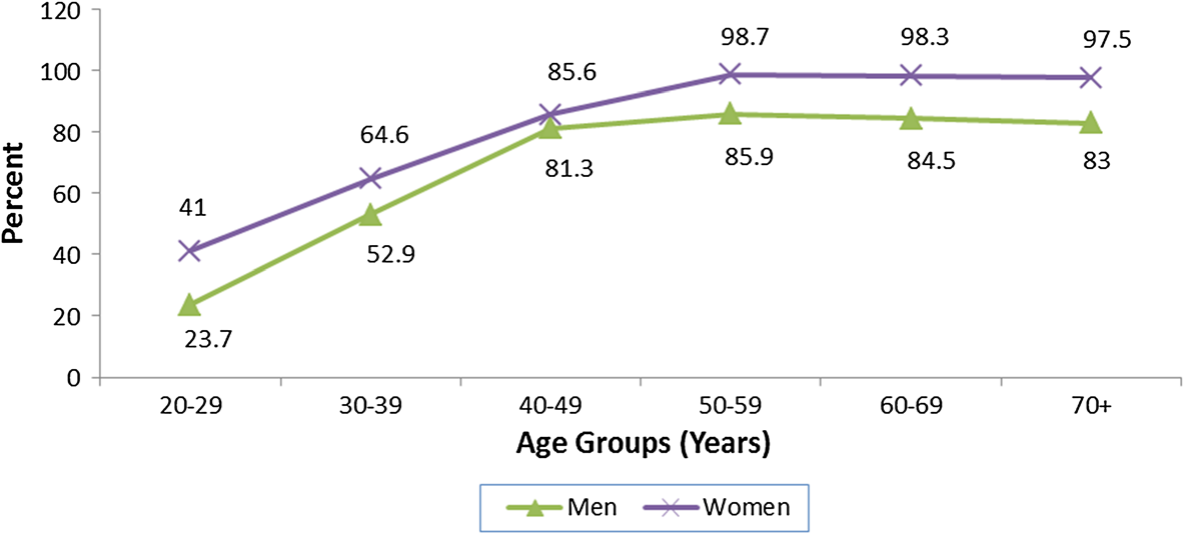
**Percent distribution of subjects with elevated %BF by age and gender.** Cut-off points: ≥25% for men and ≥32% for women.

**Figure 2:**
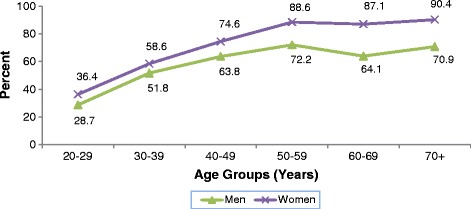
**Percent distribution of subjects with elevated WC by age and gender.** Cut-off points: ≥ 94 cm for men and ≥ 80 cm for women.

Figures 1 and 2 present the percent distribution of subjects with elevated percent body fat and elevated waist circumference respectively, distributed by age and gender. Both men and women showed rising trends in both characteristics with increasing age, with prevalence rates peaking at the age group 50–59 years. Compared with men, women presented a statistically significant higher overall proportion of elevated waist circumference (61.9% vs. 52.2% for women and men respectively, P < 0.05) and elevated %BF (69.2% vs. 58.5% for women and men respectively, P < 0.05).

### Correlates of obesity

Results of the regression analysis are presented stratified by age (20 to 59 years and ≥ 60 years) for both genders (Table 3). Importantly, the final subsample (n = 1938) included in this analysis maintained the socio-demographic characteristics of the original sample (data not shown and available upon request). In men aged 20–59 years, the odds of being obese were significantly higher among the married (OR = 2.32; 95% CI 1.46, 3.71) and those reporting family history of obesity (OR = 1.66; 95% CI 1.16, 2.37). The risk of obesity was significantly increased among men consuming >11297KJ/d (OR = 1.56; 95% CI 1.01, 2.44), and decreased among those reporting a regular meal pattern (OR = 0.68; 95% CI 0.48, 0.98). Among older men (≥60 years), family history of obesity was significantly associated with obesity (OR = 2.44; 95% CI 1.15, 5.18). Among women 20–59 years, obesity increased consistently with increasing age. The odds of being obese were lower among those with two indicators of high socioeconomic status, namely high education (OR = 0.54; 95% CI 0.32, 0.91) and low crowding index (OR = 0.62; 95% CI 0.39, 0.98). Moderate to high physical activity levels were associated with lower odds of obesity (OR = 0.66; 95% CI 0.44, 0.99), and higher energy consumption (≥8368KJ/day) was associated with higher odds (OR = 2.03; 95% CI 1.24, 3.34). Among older women (≥60 years), obesity risk decreased significantly among those who reported having a higher socioeconomic status as evaluated by household assets (OR = 0.26; 95% CI 0.10, 0.72), and being physically active (OR = 0.38; 95% CI 0.18, 0.80). Similar to men, family history of obesity showed a positive significant association with obesity in both age strata in women.

**Table 3:** **Associations of socio-demographic, lifestyle and dietary factors with obesity among Lebanese men and women, stratified by age**

**Variable (Reference category)**	**Men**		**Women**	
	**Age groups**		**Age groups**	
	**20 to 59 years (n = 742)**	**≥60 years (n = 169)**	**20 to 59 years (n = 871)**	**≥60 years (n = 156)**
	**OR (95% CI)**	**OR (95% CI)**	**OR (95% CI)**	**OR (95% CI)**
**Age (years)**				
20 to 29	1.00		1.00	
30 to 44	0.80 (0.47, 1.36)		4.25* (2.24, 8.05)	
45 to 59	1.98* (1.21, 3.52)		12.05* (6.18, 23.49)	
60 to 69		1.00		1.00
≥70		1.38 (0.63, 3.01)		1.09 (0.48, 2.43)
**Marital status**				
Never married	1.00	1.00	1.00	1.00
Ever married	2.32* (1.46, 3.71)	3.05 (0.90, 10.35)	1.20 (0.74, 1.93)	0.88 (0.40, 1.92)
**Education**				
Elementary & lower	1.00	1.00	1.00	1.00
Intermediate & higher	1.01 (0.61, 1.68)	0.59 (0.27, 1.29)	0.54* (0.32, 0.91)	0.87 (0.38, 2.02)
**Household assets**				
<7 items	1.00	1.00	1.00	1.00
≥7 items	1.44 (0.98, 2.11)	1.65 (0.70, 3.92)	0.87 (0.57, 1.33)	0.26* (0.10, 0.72)
**Crowding index**				
≥1 person/room	1.00	1.00	1.00	1.00
<1 person/room	1.14 (0.77, 1.66)	1.57 (0.70, 3.52)	0.62* (0.39, 0.98)	0.63 (0.29, 1.37)
**Physical activity**				
Low	1.00	1.00	1.00	1.00
Moderate-high	0.92 (0.65, 1.31)	1.17 (0.56, 2.44)	0.66* (0.44, 0.99)	0.38* (0.18, 0.80)
**Smoking**				
No	1.00		1.00	1.00
Yes	0.89 (0.61, 1.30)	0.64 (0.30, 1.36)	1.21 (0.82, 1.79)	0.57 (0.26, 1.21)
**Family history of obesity**				
No	1.00	1.00	1.00	1.00
Yes	1.66* (1.16, 2.37)	2.44* (1.15, 5.18)	2.77* (1.86, 4.13)	4.13* (1.84, 9.27)
**Meals pattern**				
Skips one or more meals	1.00	1.00	1.00	1.00
Eats 3 regular meals	0.68* (0.48, 0.98)	1.80 (0.76, 4.24)	0.67 (0.45, 1.01)	0.62 (0.28, 1.36)
**Food energy (KJ)**				
<8368	1.00		1.00	
8368-11297	1.08 (0.69, 1.70)		1.64* (1.02, 2.62)	
>11297	1.56* (1.01, 2.44)		2.03* (1.24, 3.34)	
**Food energy (KJ)**				
<6694		1.00		1.00
6694- 9205		1.23 (0.49, 3.06)		1.01 (0.37, 2.77)
>9205		0.93 (0.35 to 2.46)		0.82 (0.33, 2.04)
**% Food energy from fat**				
<32%	1.00	1.00	1.00	1.00
32-42%	1.06 (0.68, 1.63)	0.81 (0.32, 2.03)	1.17 (0.72, 1.90)	0.87 (0.36, 2.12)
>42%	1.24 (0.77, 1.96)	0.84 (0.32, 2.24)	1.16 (0.70, 1.91)	0.99 (0.29, 1.37)

*p < 0.05.

## Discussion

Based on a nationally representative sample of Lebanese adults, this paper reports on the prevalence of obesity (classes I, II and III), abdominal obesity, and elevated percent body fat among this population group with a focus on gender differences. It also shows a significant indication of socioeconomic differentials by obesity among women, notably for education, crowding index, and household assets, net of the effect of other potential confounding.

The findings of this study indicate an alarming increase in total mean BMI in Lebanese adults. Compared with mean BMI values determined in the previous national study carried out in 1997 [8], total mean BMI upturn ranges between 1.6 Kg/m^2^ in women {from 25.5 (SD 5.2) to 27.1 (SD 5.8)} and 2 Kg/m^2^ in men {from 25.5 (SD 4.3) to 27.5 (SD 4.7)}, exceeding the reported worldwide estimates of 0.5 Kg/m^2^ per decade for women and 0.4 Kg/m^2^ per decade for men [3]. These values remain smaller compared to weight gain estimates of 1.78 Kg/m^2^ and 1.16 Kg/m^2^ documented in Kuwaiti men and women respectively, within a shorter period of 4 years [4,10].

Of concern among the Lebanese adult population is the reported 1.5 fold increase in obesity prevalence (17% in 1999 to 26.1% in 2009), with a steeper rise among men than among women (% change between 1997 and 2009 prevalence estimates: 45.8% in men and 27.4% in women) [10]. This trend in obesity prevalence has been reported in neighbouring countries such as Iran (13.6% in 1999 to 22.3% in 2007) [13], as well as in several countries of the Arabian Gulf [4]. Within a decade, obesity rates nearly doubled among Emirati adults (16 to 34%), and increased by approximately 1.4 folds in Kuwaiti and Saudi adults (39.5 to 52% & 29.5 to 40%, respectively) [4].

This study notes that the increase in obesity prevalence among Lebanese adults has been consistent across all obesity classes, but most noticeable in obesity class III (1.8, 2, and 7 folds increase in obesity class I, II, and III respectively for men; 1.2, 1.5, and 3 folds increase in obesity class I, II, and III respectively for women). Such a rising trend, of clinical obesity in particular, within a decade entails adverse health consequences, especially if it were to follow reported trends in countries of the Arab region, ranging from 3.2% to 4.6% in Egypt, Kuwait, and Saudi Arabia [30-33] to levels that are almost 3 times higher (12.0%) in Iraq and the United Arab Emirates [4,34]. Generally, the higher estimates of obesity that have been reported in the Arab Gulf States and North African countries could be attributed to the reduced emphasis on body size and shape among men and women, indirectly related to the traditional long and wide dress worn by both genders (Abaya for women, Dishdasha for men) [8,34]. Also, in the traditional Arab culture, strong positive cultural perceptions of body fatness as a beauty criterion in women still prevail despite urbanization [35].

In accordance with data from countries in sub-Saharan Africa, South America, and the Middle East [36,37], women in the current study exhibited higher prevalence of elevated %BF and elevated WC than men. In contrast, few developed countries reported higher estimates of both characteristics in men as compared with women [38]. As BMI alone neither distinguishes fat from lean tissue nor represents adiposity directly [39], the concurrent estimates of mean BMI, mean %BF, and mean WC (cm) shown in this study indicate the prevalence of adiposity, mainly abdominal obesity, among this study population.

Our findings of an inverse relationship between obesity and socioeconomic indicators, including education, household assets, and crowding index in women, are similar to results of most studies conducted in developed countries such as Spain [40] and the United States [41], and a few developing countries and countries in nutrition transition such as Korea [42] and Iran [43].

This study certainly showed that in Lebanese women, but not in men, a better socioeconomic status may be playing a protective role in the likelihood of obesity through manipulation of diet and/ or physical activity away from positive energy balance. It has been noted that gender differences in obesity and socioeconomic status may be attributed to a constellation of psychosocial and behavioural factors including dietary intake and physical activity [41]. A better economic standing may prevent obesity in terms of the resources available to buy food and to participate in leisure time physical activities; whereas education, associated with the acquisition of beliefs and knowledge, enables people to integrate healthy behaviours into a coherent lifestyle [44]. The lack of association between socioeconomic indicators and obesity among men may be attributed to the different attitudes toward body weight status across gender [45]. Thus, women are more likely to use resources, whether economic, cultural or social, to shift their diet and activity patterns in pursuit of a healthier body weight than men, where the increase in earnings and purchasing power does not necessarily lead to a healthier lifestyle [43,46,47].

In the current study, marriage was significantly predictive of obesity for men of both age strata, but not for women. Many known or unknown socio-demographic factors influence the relationships between diet/ food choices, physical activity and body weight, and when linked together, may or may not lead to obesity in specific population groups [43,48]. Studies examining gender differences in the marriage-obesity relationship reported that married women were less likely to be obese compared with married men, and in certain ethnic groups, women showed no association between marriage and body weight [49,50]. The marriage-obesity relationship may vary with age, gender, and ethnicity, but the exact mechanism linking these variables is not fully understood [50]. In young cohorts, research attributed this association to entry into a relationship and sharing the same household environment that may in turn influence partners’ food choices and eating habits [51,52]. Furthermore, entry into marriage has been associated with decreased physical activity, paralleled with increased social obligations promoting increased food intake and energy consumption [52,53].

In comparison with the 2010 dietary guidelines for Americans [29], the presented data indicate that the energy consumption level significantly increasing the risk of obesity among Lebanese adults is at the higher limit of recommended intake at moderate activity levels for both genders.

Examining the diet composition of this population, this study showed mean percent energy consumption from fat exceeding the 30% limit recommended by the WHO [54], as well as the 35% limit recommended by the institute of medicine [55] in both genders; whereas intakes of carbohydrates and proteins were at the lower limit of the recommended intake. Evidence shows that in terms of energy balance and body weight maintenance, the critical issue is not the relative proportion of macronutrients in the diet but rather the total energy consumed per day. It has been reported that a relatively modest change in the overall energy density of the diet of a person consuming a consistent weight of food would significantly impact his or her daily energy consumption [56]. Because of its high energy content of 37.7KJ per gram, fat influences energy density values of foods more than carbohydrate or protein (16.8KJ per gram) [56,57]. Also, scientists reported that subjects in experimental situations readily overeat when presented with high-fat foods. This has been attributed to the weak satiating capacity of fat [58]. Acceptable macronutrients distribution in the diet, along with quality choices of unsaturated fats, unrefined cereals, and plant-based or animal-based high biological value proteins, ought to be maintained to reduce metabolic complications and chronic disease risks [29,57,59,60].

On the other side of the energy balance equation is energy expenditure of which physical activity is a major component [18]. In many communities, women may face barriers that limit their access to and participation in outdoor physical activities and sports [43]. In most of the Gulf Cooperation Council Countries, and in certain communities of different ethnic/religious backgrounds in Lebanon, segregation of men and women is favoured, and women-only exercise facilities are rare and highly expensive [31]. Furthermore, in certain areas across the Governorates of Lebanon, mainly rural villages, it is also socially unacceptable for women to walk or exercise alone outside the house without the company of a family member [31]. This study provides further evidence on the likely effect of those social factors on obesity prevalence among women of lower education and socioeconomic status.

The observed significantly negative relationship between obesity and consistent meal consumption pattern in men merits discussion. Clinical studies document that regular meal consumption can potentially reduce the risk of obesity and chronic diseases through mechanisms involved in energy balance and metabolism [61]. In a study carried out in healthy lean women, irregular meal frequency led to a lower postprandial energy expenditure compared with the regular meal frequency, while the mean energy intake was not significantly different between the two. Clinical trials reported that the reduced thermogenic effect of food with the irregular meal frequency may lead to weight gain in the long term [62].

Finally, family history of obesity remains a major predictive factor of obesity in both genders and age groups. This probably indicates that not only shared genes, but also behaviours, lifestyles, and environments within families could possibly increase their risk of developing health problems including obesity [63].

The findings of this study should be considered in light of its limitations and offsetting strengths. The cross-sectional nature of the study does not allow inferences to be drawn with respect to the causal relationship between dependent and certain independent variables. The proxy socioeconomic indicator “household assets” was based on reported asset ownership and this may not provide reliable and valid assessment of the overall socioeconomic position of the individual as would those obtained from direct measurements of income and expenditure (when reliably collected) [64]. Days of the week effects on dietary intake could not be completely accounted for in the study due to disproportionate over-representation of working days, which is likely to have underreported the extent of overall dietary intake at the national level. Nevertheless, the study has been carried out during the months of May 2008 till April 2009, thus allowing the researcher to trace monthly variations in dietary intake. Importantly, implausible reporters of energy intake were identified and excluded from the analysis, thus improving the validity of the association between diet and weight status. The exclusion of participants with missing data on pertinent variables may have introduced selection biases; yet, the final sample included in the analysis was comparable in terms of demography and geography to the original one.

It is essential to note that the data collection involved face-to-face interviews at the participant’s home, a setting that may possibly contribute to socially desirable responses and information bias [65]. In particular, under-reporting of food intake and overestimation of self-reported physical activity levels have been recognized in many studies [66,67]. Hence, interviewer refresher training sessions to reinforce effective probing skills were regularly carried out during the data collection period. Also, anthropometric measures were determined by trained nutritionists using calibrated instruments thus ruling out the possibility of reported underestimation or overestimation of weight and height, and consequently BMI. The dietary assessment tool was implemented by trained nutritionists and using standardized procedures.

For homogeneity purposes, BMI was used as the main outcome of the current study for the assessment of obesity and its correlates across gender and age groups. While BMI does appear to have excellent validity as a measure of absolute fat mass adjusted for height in young and middle-aged adults, it has its limitations in detecting the physiological age-related transformations of lean mass to fat mass in older adults [68,69]. Thus, waist circumference might have been a better predictor of adiposity than BMI in older adults [69]. Nevertheless, the findings of the current study shed light on the prevalence and correlates of obesity among the elderly, showing the need for further in-depth research on the risk factors for obesity among older adults, taking into consideration diet and body composition changes as well as social and health conditions.

## Conclusion

The study findings underscore the importance of dietary, lifestyle, and socioeconomic determinants of obesity, and highlight gender disparities in these associations in an adult population living in a country experiencing nutrition transition.

Taking the high risk approach, culturally-appropriate, community-based interventions should be initiated to help increase nutrition awareness among specific population segments, especially women of low education and socioeconomic levels. Understanding the importance of a healthy balanced diet and physical activity in the prevention of lifestyle-related diseases, regardless of genetic susceptibility, is crucial. Nutrition awareness campaigns are indeed recommended; however, strategies to improve the environments within which individual behavioural decisions are made should be considered, mainly those related to food availability, cost of healthy foods, and access to physical activity opportunities. Most important is the delivery of a stable food security level for all the households in the six Governorates of Lebanon.

Lifestyle factors are policy-relevant elements and hence, policy makers and commissioners of health services who are responsible for public health should tailor their efforts and resources to tackling obesity in light of its dynamics in the Lebanese adult population.

## References

[1] Rokholm B, Baker JL, Sorensen TI (2010). The leveling off of the obesity epidemic since the year 1999: a review of evidence and perspectives. Obes Rev.

[2] Popkin BM (2010). Recent dynamics suggest selected countries catching up to US obesity. Am J Clin Nutr.

[3] Finucane MM, Stevens GA, Cowan MJ, Danaei G, Lin JK, Paciorek CJ (2011). National, regional, and global trends in body-mass index since 1980: systematic analysis of health examination surveys and epidemiological studies with 960 country-years and 9.1 million participants. Lancet.

[4] Ng SW, Zaghloul S, Ali HI, Harrison G, Popkin BM (2011). The prevalence and trends of overweight, obesity and nutrition-related non-communicable diseases in the Arabian Gulf states. Obes Rev.

[5] World Health Organization. Draft nutrition strategy and plan of action for countries of the Eastern Mediterranean Region 2010–2019. WHO Regional Office for the Eastern Mediterranean; 2009. www.emro.who.int/docs/EM_RC57_4_en.pdf?ua=1.

[6] Ministry of Health (2006). The Epidemiological Surveillance Program and Department of Statistics. Population Estimates in Lebanon.

[7] World Statistics Pocketbook. Country Profile: Lebanon. United Nations Statistics Division; 2011. http://data.un.org/CountryProfile.aspx?crName=Lebanon.

[8] Sibai AM, Hwalla N, Adra N, Rahal B (2003). Prevalence and covariates of obesity in Lebanon: findings from the first epidemiological study. Obes Res.

[9] Sibai AM, Obeid O, Batal M, Adra N, El Khoury D, Hwalla N (2008). Prevalence and correlates of metabolic syndrome in an adult Lebanese population. Prev Control.

[10] Hanson KL, Sobal J, Frongillo EA (2007). Gender and marital status clarify associations between food insecurity and body weight. J Nutr.

[11] Naja F, Nasreddine L, Itani L, Chamieh MC, Adra N, Sibai AM (2011). Dietary patterns and their association with obesity and socio-demographic factors in a national sample of Lebanese adults. Public Health Nutr.

[12] World Health Organization (2005). WHO STEPS Surveillance Manual: The WHO STEP wise approach to chronic disease risk factor surveillance.

[13] Esteghamati A, Khalilzadeh O, Mohammad K, Meysamie A, Rashidi A, Kamgar M (2010). Secular trends of obesity in Iran between 1999 and 2007: national surveys of risk factors of non-communicable diseases. Metab Syndr Relat Disord.

[14] IPAQ Research Committee (2005). Guidelines for Data Processing and Analysis of the International Physical Activity Questionnaire (IPAQ) - Short and Long Forms.

[15] World Health Organization: Obesity: Preventing and managing the global epidemic (2000). Report of a WHO consultation (WHO Technical Report Series 894).

[16] World Health Organization: Recommended Measurement Protocols and Derivation of Indices (1995). Physical Status: The Use and Interpretation of Anthropometry (pp 424–438).

[17] Durnin JV, Womersley J (1974). Body fat assessed from total body density and its estimation from skinfold thickness: measurements on 481 men and women aged from 16 to 72 years. Br J Nutr.

[18] Lee RD, Nieman DC (2010). Nutritional assessment.

[19] Zimmet P, Magliano D, Matsuzawa Y, Alberti G, Shaw J (2005). The metabolic syndrome: a global public health problem and a new definition. J Atheroscler Thromb.

[20] Moshfegh AJ, Rhodes DG, Baer DJ, Murayi T, Clemens JC, Rumpler WV (2008). The US department of agriculture automated multiple-pass method reduces bias in the collection of energy intakes. Am J Clin Nutr.

[21] Thompson FE, Subar AF, Coulston AM, Boushey CJ (2008). Dietary assessment methodology. Nutrition in the prevention and treatment of disease.

[22] Nasreddine L, Sibai A, Mrayati M, Adra N, Hwalla N (2009). Adolescent obesity in Syria: prevalence and associated factors. Child Care Health Dev.

[23] Pellet PL, Shadarevian S (1970). Food Composition Tables for use in the Middle East.

[24] Prakongsai P (2006). An application of the asset index for measuring household living standards in Thailand.

[25] Galobardes B, Shaw M, Lawlor DA, Lynch JW, Davey Smith G (2006). Indicators of socioeconomic position. J Epidemiol Community Health.

[26] Lebanese Republic Ministry of Social Affairs (2006). Central Administration for Statistics and UNDP: Living Conditions of households - The National Survey of Household Living Conditions 2004:106–107.

[27] Black AE (2000). Critical evaluation of energy intake using the Goldberg cut-off for energy intake: Basal metabolic rate. A practical guide to its calculation, use and limitations. Int J Obes Relat Metab Disord.

[28] Livingstone MB, Black AE (2003). Markers of the validity of reported energy intake. J Nutr.

[29] United States Department of Agriculture & United States Department of Health and Human Services (2010). Report of the Dietary Guidelines Advisory Committee on the Dietary Guidelines for Americans, 2010.

[30] Galal OM (2002). The nutrition transition in Egypt: obesity, undernutrition and the food consumption context. Public Health Nutr.

[31] Sibai AM, Nasreddine L, Mokdad AH, Adra N, Tabet M, Hwalla N (2010). Nutrition transition and cardiovascular disease risk factors in Middle East and North Africa countries: reviewing the evidence. Ann Nutr Metab.

[32] Al-Kandari YY (2006). Prevalence of obesity in Kuwait and its relation to sociocultural variables. Obes Rev.

[33] Al-Nozha MM, Al-Mazrou YY, Al-Maatouq MA, Al-Nozha MM, Al-Mazrou YY, Al-Maatouq MA (2005). Obesity in Saudi Arabia. Saudi Med J.

[34] Al-Tawil NG, Abdulla MM, Abdul Ameer AJ (2007). Prevalence of and factors associated with overweight and obesity among a group of Iraqi women. East Mediterr Health J.

[35] Mousa T, Al-Domi H, Mashal R (2009). Eating disturbances in adolescent girls: a review. Dirasat Agr Sci.

[36] Fezeu L, Minkoulou E, Balkau B, Kengne AP, Awah P, Unwin N (2006). Association between socioeconomic status and adiposity in urban Cameroon. Int J Epidemiol.

[37] Al-Lawati JA, Jousilahti PJ (2004). Prevalence and 10-year secular trend of obesity in Oman. Saudi Med J.

[38] Rodriguez E, Lope B, Lopez AM, Ortega RM (2011). Overweight and obesity among Spanish adults. Nutr Hosp.

[39] Flegal KM, Carroll MD, Ogden CL, Curtin LR (2010). Prevalence and trends in obesity among US adults, 1999–2008. JAMA: J Am Med Dir Assoc.

[40] Garcia-Alvarez A, Serra-Majem L, Ribas-Barba L, Castell C, Foz M, Uauy R (2007). Obesity and overweight trends in Catalonia, Spain (1992–2003): gender and socio-economic determinants. Public Health Nutr.

[41] Sanchez-Vaznaugh EV, Kawachi I, Subramanian SV (2009). Do socioeconomic gradients in body mass index vary by race/ethnicity, gender, and birthplace?. Am J Epidemiol.

[42] Yoon YS, Oh SW, Park HS (2006). Socioeconomic status in relation to obesity and abdominal obesity in Korean adults: a focus on sex differences. Obesity (Silver Spring, Md).

[43] Hajian-Tilaki KO, Heidari B (2010). Association of educational level with risk of obesity and abdominal obesity in Iranian adults. J Public Health.

[44] Assi F, Devaux M, Church J, Cecchini M, Borgonovi F. Education and Obesity in Four OECD Countries. OECD Publishing Papers. http://www.olis.oecd.org/olis/2009doc.nsf/LinkTo/N.

[45] Giskes K, Turrell G, van Lenthe FJ, Brug J, Mackenbach JP (2006). A multilevel study of socio-economic inequalities in food choice behaviour and dietary intake among the Dutch population: the GLOBE study. Public Health Nutr.

[46] Zhang Q, Wang Y (2004). Socioeconomic inequality of obesity in the United States: do gender, age, and ethnicity matter?. Soc Sci Med.

[47] Reynolds SL, Hagedorn A, Yeom J, Saito Y, Yokoyama E, Crimmins EM (2008). A tale of two countries-the United States and japan: are differences in health due to differences in overweight?. J Epidemiol.

[48] Swinburn BA, Sacks G, Hall KD, McPherson K, Finegood DT, Moodie ML (2011). The global obesity pandemic: shaped by global drivers and local environments. Lancet.

[49] Sobal J, Hanson KL, Frongillo EA (2009). Gender, ethnicity, marital status, and body weight in the United States. Obesity (Silver Spring, Md).

[50] Tzotzas T, Vlahavas G, Papadopoulou SK, Kapantais E, Kaklamanou D, Hassapidou M (2010). Marital status and educational level associated to obesity in Greek adults: data from the national epidemiological survey. BMC Public Health.

[51] Gordon-Larsen P, The NS (2009). Entry into romantic partnership is associated with obesity. Obesity (Silver Spring, Md).

[52] Bell S, Lee C (2005). Emerging adulthood and patterns of physical activity among young Australian women. Int J Behav Med.

[53] Burke V, Beilin LJ, Dunbar D, Kevan M (2004). Changes in health-related behaviours and cardiovascular risk factors in young adults: Associations with living with a partner. Prev Med.

[54] World Health Organization (2003). Diet, Nutrition and the Prevention of Chronic Diseases. Report of the joint WHO/FAO expert consultation. (WHO Technical Report Series 916).

[55] Dietary Reference Intakes (DRIs) (2002). Dietary Reference Intakes for Energy, Carbohydrate, Fiber, Fat, Fatty Acids, Cholesterol, Protein, and Amino Acids (2002/2005).

[56] Rolls BJ (2009). The relationship between dietary energy density and energy intake. Physiol Behav.

[57] Drewnowski A (2007). The real contribution of added sugars and fats to obesity. Epidemiol Rev.

[58] Kopelman PG (2000). Obesity as a medical problem. Nature.

[59] Hall KD, Sacks G, Chandramohan D, Chow CC, Wang YC, Gortmaker SL (2011). Quantification of the effect of energy imbalance on bodyweight. Lancet.

[60] Skidmore P (2007). Macronutrient intakes and their role in obesity. Nutr Bull.

[61] Maureen T, Mark A (2007). Breakfast frequency and quality in the etiology of adult obesity and chronic diseases. Nutr Rev.

[62] Farshichi HR, Taylor MA, Macdonald IA (2004). Decreased thermic effect of food after an irregular compared with a regular meal pattern in healthy lean women. Int J Obes.

[63] Center of Disease Control Database. [www.cdc.gov/features/familyhealthhistory]

[64] Howe LD, Hargreaves JR, Gabrysch S, Huttly SR (2009). Is the wealth index a proxy for consumption expenditure? A systematic review. J Epidemiol Community Health.

[65] Yu IT, Tse SL (2012). Clinical epidemiology workshop 4-Sources of bias in case-referent studies. Hong Kong Med J.

[66] Mendez MA, Popkin BM, Buckland G, Schroder H, Amiano P, Barricarte A (2011). Alternative methods of accounting for underreporting and overreporting when measuring dietary intake-obesity relations. Am J Epidemiol.

[67] Maddison R, Ni Mhurchu C, Jiang Y, Vander Hoorn S, Rodgers A, Lawes CM (2007). International physical activity questionnaire (IPAQ) and New Zealand physical activity questionnaire (NZPAQ): a doubly labelled water validation. Int J Behav Nutr Phys Act.

[68] World Health Organization (2008). Expert Consultation-Geneva.

[69] Zamboni M, Mazzali G, Zoico E, Harris TB, Meigs JB, Di Francesco V (2005). Health consequences of obesity in the elderly: a review of four unresolved questions. Int J Obes (Lond).

